# Synergistic Biocontrol and Growth Promotion in Strawberries by Co-Cultured *Trichoderma harzianum* TW21990 and *Burkholderia vietnamiensis* B418

**DOI:** 10.3390/jof10080551

**Published:** 2024-08-05

**Authors:** Wenzhe Li, Yiting Fu, Yanqing Jiang, Jindong Hu, Yanli Wei, Hongmei Li, Jishun Li, Hetong Yang, Yuanzheng Wu

**Affiliations:** 1 School of Bioengineering, Qilu University of Technology, Shandong Academy of Sciences, Jinan 250353, China; lwenzhe1008@163.com (W.L.); fuyiting0525@163.com (Y.F.); yanqing597976@163.com (Y.J.); 2 Shandong Provincial Key Laboratory of Applied Microbiology, Ecology Institute, Qilu University of Technology, Shandong Academy of Sciences, Jinan 250103, China; hujd@sdas.org (J.H.); yanli_wei@163.com (Y.W.); hmlihm@163.com (H.L.); yewu2@sdas.org (J.L.); yanght@sdas.org (H.Y.)

**Keywords:** secondary metabolites, metabolomic profiles, co-culture, *Trichoderma harzianum*, *Burkholderia vietnamiensis*, *Colletotrichum siamense*, control efficiency

## Abstract

This study aimed to investigate the efficiency of the secondary metabolites (SMs) produced by a co-culture of *Trichoderma harzianum* TW21990 and *Burkholderia vietnamiensis* B418 in the control of *Colletotrichum siamense* CM9. A fermentation filtrate of B418 + TW21990 co-culture (BT21) produced a notable increase in the inhibition rate of CM9 compared to those of TW21990 and B418 monocultures, which reached 91.40% and 80.46% on PDA plates and strawberry leaves, respectively. The BT21 fermentation broth exhibited high control efficiency on strawberry root rot of 68.95% in a pot experiment, which was higher than that in the monocultures and fluazinam treatment. In addition, BT21 treatment promoted strawberry root development, improved antioxidative enzyme activities in the leaves and roots, and enhanced the total chlorophyll content of the strawberry leaves. UHPLC-MS/MS analysis of fermentation filtrates was performed to elucidate SM variations, revealing 478 and 795 metabolites in BT21 co-culture in positive and negative ion modes, respectively. The metabolomic profiles suggested abundant SMs with antagonistic capabilities and growth-promoting effects: 3-(propan-2-yl)-octahydropyrrolo [1,2-a]pyrazine-1,4-dione (cyclo(L-Pro-L-Val)), 3-[(4-hydroxyphenyl)methyl]-octahydropyrrolo[1,2-a]pyrazine-1,4-dione (cyclo(L-Pro-L-Tyr)), 3-indoleacetic acid (IAA), 2-hydroxycinnamic acid, 4-aminobutyric acid (GABA), bafilomycin B1, and DL-indole-3-lactic acid (ILA) were significantly enhanced in the co-culture. Overall, this study demonstrates that a co-culture strategy is efficient for inducing bioactive SMs in *T. harzianum* and *B. vietnamiensis,* which could be exploited as a novel approach for developing biocontrol consortia.

## 1. Introduction

*Trichoderma* spp. are soil-borne filamentous fungi and have been well recognized as biocontrol agents (BCAs) owing to their antagonistic effects against phytopathogens [1]. The genus *Trichoderma* is widely distributed in the soil, root ecosystems, and plant debris, which can rapidly colonize substrates, improve plant growth, increase disease resistance, and antagonize different phytopathogens [2]. The antagonistic activities of *Trichoderma* spp. include direct and indirect mechanisms depending on the strain and species. The direct mechanisms involve the production of lytic enzymes, active metabolites, and mycoparasitism, whereas the indirect mechanisms involve competition for nutrients and space, induction of plant defenses, and promotion of plant growth [3,4]. Moreover, *Trichoderma* spp. can produce diverse metabolites including pyrones, trichothecenes, sesquiterpenes, cyclopeptides, diketopiperazines, alkaloids, polyketides, and peptaibols, which not only inhibit the development of pathogens but also trigger the defense system of host plants [5].

The *Burkholderia* genus belongs to the Betaproteobacteria class, which occupies a remarkably wide range of ecological niches [6]. Although the pathogenic group of *Burkholderia* spp. can cause animal, human, and plant diseases including *Burkholderia cepacia* complex (Bcc), the nonpathogenic members of *Burkholderia* species are receiving increasing attention as plant-growth-promoting rhizosphere bacteria (PGPR) owing to their multiple functions in bioremediation, the biocontrol of phytopathogens, and growth promotion [7,8]. *Burkholderia* spp. can produce an arsenal of metabolites, such as pyrrolnitrins, phenazines, diketopiperazines, lipopeptides, quinolones, polyketides, and siderophores, to increase their rhizosphere competence [9,10]. For instance, *B. vietnamiensis* displayed the ability to fix nitrogen and generate siderophore, indole-3-acetic acid (IAA), and 1-aminocyclopropane-1-carboxylate (ACC) deaminase in the rhizosphere and rhizoplanes of maize, barley, and sorghum plants [11]. Numerous members of *Burkholderia* spp. have been exploited as agricultural inoculants, biocontrol agents, and biofertilizers for agricultural applications [12].

Secondary metabolites (SMs) are a class of low-molecular-weight compounds produced by microorganisms, which are not essential for microbial growth and have a variety of biological activities [13]. SMs play a crucial role during microbial interactions as competitive weapons, metal-transporting agents, symbiotic agents between microorganisms, and signaling molecules for intra/interspecies chemical communication [14]. Various SMs like antibiotics, alkaloids, terpenoids, flavonoids, lipopeptides, and polyketides not only modulate the responses of individuals and populations but also shape entire microbial communities [15,16]. With the development of genomic sequencing, abundant biosynthetic gene clusters (BGCs) putative for SM synthesis have been identified in microbial genomes [17]. However, there are large gaps between the genome capacity of BGCs and the discovered SMs in microorganisms since enormous BGCs remain silent or have low expression levels under normal laboratory culture conditions [18].

The co-culture of different microorganisms has emerged as an effective approach for stimulating the yield and variety of SMs [19]. By mimicking the natural microbial community, co-culture systems create favorable environmental conditions to activate the expression of silent BGCs and lead to the production of new SMs [20,21]. As widely studied BCAs in agriculture, *Trichoderma* and *Bacillus*, *Burkholderia*, and *Pseudomonas* (PGPR) can utilize their complementary advantages to produce synergistic effects via co-culture [22]. Velmourougane et al. verified the in vitro compatibility of *Trichoderma* and *Azotobacter* in a co-culture system to increase biomass and biofilm formation, showing antagonistic ability and enhanced SMs production [23]. Li et al. optimized the co-culture conditions of *T. atroviride* SG3403 and *Bacillus subtilis* 22 for the production of antifungal SMs; they found the fermented filtrate of co-culture inhibited *Fusarium graminearum* by 54.22% and identified mevastatin and koninginin in the co-culture via LC–MS analysis [24]. The metabolomics approach provides an efficient and accurate detection technique and analytical method to systematically determine the interactions and changes among microorganisms [25]. The combination of co-culture systems with metabolomic profiles will accelerate the identification and quantification of bioactive SMs [26].

In this context, we employed a co-culture strategy to increase the production of SMs by *Trichoderma harzianum* TW21990 and *Burkholderia vietnamiensis* B418, which have been characterized as elite BCAs and displayed antifungal abilities against *Colletotrichum siamense* CM9, which causes root rot in strawberry [27,28]. To the best of our knowledge, there are no previous reports regarding a *Trichoderma* and *Burkholderia* co-culture system. The objective of this study was to investigate the antagonistic activities and growth-promoting effects of the SMs produced by mono- and co-cultures of TW21990 and B418, to elucidate SM differences among different culture modes using metabolomic profiles, and to provide a theoretical basis and data support for the development of BCA consortia.

## 2. Materials and Methods

### 2.1. Microorganisms and Plant Species

*Trichoderma harzianum* TW21990 (China General Microbiological Culture Collection Center, CGMCC No. 12864) and *Burkholderia vietnamiensis* B418 (CGMCC No. 1212) were isolated and preserved by the Environmental Microbiology Laboratory, Institute of Ecology, Shandong Academy of Sciences. *Colletotrichum siamense* CM9 was isolated from the soil of strawberry greenhouse in Jinan that was experiencing root rot. The strawberry (*Fragaria* × *ananassa* Duch.) cv. ‘Hongyan’ used in this study was the same as previously planted in that greenhouse, purchased from Tai’an Renyuan Seeds Co., Ltd (Tai’an, China).

### 2.2. Medium and Cultivation Conditions

*T. harizanum* TW21990 was cultivated in 9 cm Petri dishes containing potato dextrose agar (PDA; peeled potato 200 g L^−1^, dextrose 20 g L^−1^, agar 15 g L^−1^, distilled water 1000 mL) for 5 days at 28 °C. The spores were washed with sterile distilled water (SDW) to a final concentration of 2 × 10^8^ cfu/mL and used as the inoculum for culture.

*B. vietnamiensis* B418 was cultivated on tryptone-yeast extract medium (TY; peptone 10 g L^−1^, yeast powder 1 g L^−1^, CaCl_2_ 0.2 g L^−1^, agar 15 g L^−1^, distilled water 1000 mL, pH 7.2–7.4) for 1–2 days at 30 °C. To prepare the seed culture, a single colony of B418 was streaked and inoculated into TY broth, the culture was cultivated at 30 °C and 180 rpm for 24 h, and the concentration was adjusted to 2 × 10^8^ cfu/mL with SDW.

For co-culture, 200 μL of *T. harizanum* TW21990 spore suspension and an equivalent bacterial suspension of *B. vietnamiensis* B418 were inoculated into 200 mL of modified King’s B broth (MKB; casein amino acids 20 g L^−1^, glycerol 10 mL, K_2_HPO_4_ 1.5 g L^−1^, MgSO_4_·7H_2_O 1.5 g L^−1^, distilled water 1000 mL, pH 7.2 ± 0.2) in 1000 mL conical flasks. The fermentation broth was cultivated at 28 °C and 180 rpm for 7, 10, or 14 days. For the monoculture, TW21990 and B418 were inoculated into 200 mL of MKB at the same inoculation ratio and cultivated at the same conditions. The cultures were centrifuged at 12,000 × *g* for 5 min at 4 °C, and the supernatants were collected for filtration through a 0.22 μm membrane filter (Merck & Co., Inc., Rahway, NJ USA) to obtain the fermentation filtrates. The fermentation filtrates were maintained at –80 °C until further analysis.

### 2.3. Microscopic Analysis

The ultrastructures of *T. harizanum* TW21990 and *B. vietnamiensis* B418 in mono- and co-cultures were observed by scanning electron microscopy (SEM). Briefly, the cultures were fixed in 2.5% glutaraldehyde (*w*/*v*) at room temperature for 12 h, rinsed with 0.1 M phosphate buffer (pH 7.0) thrice for 15 min, and then washed with an ethanol gradient (30%, 50%, 70%, 90%, and 100% (*v*/*v*)) for 10 min for dehydration. Finally, the samples were critically dried with carbon dioxide, mounted on aluminum stubs with conductive carbon cement, and coated with palladium via magnetron sputtering. All samples were examined using a scanning electron microscope (Hitachi Regulus SU8100, Hitachi Inc., Tokyo, Japan).

### 2.4. Antifungal Activity Assays

#### 2.4.1. Inhibition Activity of Fermentation Filtrates on Mycelial Growth

The growth inhibition of obtained fermentation filtrates (*T. harizanum* TW21990 and *B. vietnamiensis* B418 in mono- and co-cultures, respectively) against *C. siamense* CM9 was evaluated using the mycelial growth rate. In brief, the fermentation filtrates were supplemented into sterilized PDA medium at different volume ratio of 5%, 10%, and 20% at 50–55 °C to make plates, while the same volume of SDW was used as a negative control. A 5 mm diameter inoculum plug of CM9 was taken from the active growing mycelial of young colonies and placed in the center of these prepared PDA plates aseptically. The growth diameter of CM9 was measured using a cross method after cultivation at 28 °C for 5 days [29]. There were five replicates in each treatment, and the assay was repeated twice. The mycelial growth inhibition rate was determined separately according to the following formula:

(1) $$\begin{array}{c} \mathrm{Mycelial}\;\mathrm{inhibition}\;\mathrm{rate}=\\ \frac{Mycelial\;diameter\;of\;Colletotrichum\;siamense\;CM9\;in\;control-Mycelial\;diameter\;of\;C.\;siamense\;CM9\;in\;treatment}{Mycelial\;diameter\;of\;Colletotrichum\;siamense\;CM9\;in\;control}\times 100\% \end{array}$$

#### 2.4.2. Antagonistic Activity of Fermentation Filtrates Using Detached Leaves

Five-week-old strawberry seedlings were used to evaluate the antagonistic activity of fermentation filtrates against *C. siamense* CM9. The leaves were cut from strawberry plants, washed with SDW, soaked in 75% ethanol for 1 min, washed again with SDW, and finally put on aseptic filter paper in 9 cm Petri dishes for air drying on a clean bench. Either 1 mL of fermentation filtrate or MKB broth and SDW (control groups) was sprayed on strawberry leaves; the plate was sealed with sealing film (Parafilm, Fisher Scientific, Waltham, MA, USA) and placed at 28 °C for 12 h for full absorption. Then, a 5 mm plug of CM9 was inoculated in the center of the leaf, and cultivate continued at 28 °C for 4 days before measuring the lesion diameters. The experiment included at least 20 leaves from 6 strawberry seedlings in each treatment, and the experiment was repeated twice.

### 2.5. Pot Experiment in Greenhouse

Five-week-old strawberry seedlings were selected and transplanted into flowerpots containing 300 g of soil for a pot experiment to verify the effect of *T. harizanum* TW21990 and *B. vietnamiensis* B418 mono- and co-cultures on the control of *C. siamense* CM9. As listed in Table 1, there were 6 treatments designed for the experiment: blank control without pathogen inoculation (CK), negative control of *C. siamense* CM9 pathogen (CM9), positive control of fungicide fluazinam with CM9 inoculated (FZN), fermentation broth of B418 monoculture with CM9 inoculation (B1), fermentation broth of TW21990 monoculture with CM9 inoculation (T21), and fermentation broth of B418 + TW21990 co-culture with CM9 inoculation (BT21). There were 12 replicates in each treatment, and the experiment was repeated twice. The mean temperature in the greenhouse was set to 28–35 °C during the day and 10–15 °C during the night, with 80%–90% relative humidity.

The incidence and disease severity of the strawberry plants in each treatment were recorded every day, and the disease index (DSI) and control effect were evaluated 30 days after inoculation. Infection severity was assessed using a disease index of grade 0 to 5, where grade 0 = no wilt, grade 1 ≤ 30% leaves wilted, grade 2 = 30–60% of leaves wilted, grade 3 = 60–80% of leaves wilted, grade 4 > 80% of leaves wilted, and grade 5 = complete collapse [30]. The disease index and control effect were determined according to the following formulas:

(2) $$\mathrm{Disease}\;\mathrm{index}=\sum \frac{Number\;of\;plants\;of\;each\;disease\;grade\times Representative\;value\;of\;each\;disease\;grade}{Total\;number\;of\;plants\times representative\;value\;of\;the\;highest\;disease\;grade}\times 100$$

(3) $$\mathrm{Control}\;\mathrm{effect}=\frac{Control\;disease\;index-treatment\;disease\;index}{Control\;disease\;index}\times 100\%$$

#### 2.5.1. Determination of Antioxidative Enzyme Activity

The leaves and roots were selected from strawberry plants the after pot experiment. A total of 0.1 g of plant tissue was accurately weighed and submerged in 1 mL of extract solution and ground in a mortar with a pestle in liquid nitrogen; the filtrate was centrifuged at 8000× *g* for 10 min at 4 °C; and the supernatant was collected the determination of different enzyme activities. Peroxidase (POD), superoxide dismutase (SOD), and catalase (CAT) activities were determined using the corresponding enzyme activity assay kits (BC0090, BC0170, and BC0200, Solarbio, Beijing, China).

#### 2.5.2. Determination of Total Chlorophyll Content

The strawberry leaves were rinsed with distilled water, the surface moisture was absorbed, and the midrib was removed. Then, 0.1 g of leaves was accurately weighed and cut into 2 mm segments, 3 mL of 80% acetone was added, then the leaves were fully ground in a mortar with a pestle in liquid nitrogen. The solution was transferred to sterile Eppendorf tubes and stored at 4 °C in the dark for one week. Total chlorophyll content was determined at wavelengths of 645 and 663 nm.

### 2.6. Metabolomic Profiles

#### 2.6.1. Metabolites Extraction

A total of 1 mL of fermentation filtrates was freeze-dried and resuspended with 100 μL of prechilled 80% methanol with a well vortex. Then, the samples were incubated on ice for 5 min and centrifuged at 15,000× *g* for 15 min at 4 °C. The supernatant was diluted to final concentration containing 53% methanol with LC–MS-grade water. The samples were subsequently transferred to a fresh Eppendorf tube and then centrifuged at 15,000× *g* for 15 min at 4 °C. Finally, the supernatant was injected into the LC–MS/MS system for analysis.

#### 2.6.2. UHPLC-MS/MS Analysis

Ultra-high-performance liquid chromatography–MS/MS (UHPLC-MS/MS) analysis was performed using a Vanquish UHPLC system (Thermo Fisher, Bremen, Germany) coupled with an Orbitrap Q Exactive™ HF-X mass spectrometer (Thermo Fisher, Germany) at Novogene Co., Ltd. (Beijing, China). Samples were injected onto a Hypesil Gold column (100 × 2.1 mm, 1.9 μm) using a 17 min linear gradient at a flow rate of 0.2 mL/min. The eluents for the positive polarity mode were eluent A (0.1% formic acid in water) and eluent B (methanol). The eluents for the negative polarity mode were eluent A (5 mM ammonium acetate, pH 9.0) and eluent B (methanol). The solvent gradient was set as follows: 2% B, 1.5 min; 2–85% B, 3 min; 85–100% B, 10 min; 100–2% B, 10.1 min; 2% B, 12 min. The Q Exactive™ HF-X mass spectrometer was operated in positive/negative polarity mode with spray voltage of 3.5 kV, capillary temperature of 320 °C, sheath gas flow rate of 35 psi, aux gas flow rate of 10 L/min, S-lens RF level of 60, and Aux gas heater temperature of 350 °C.

#### 2.6.3. Data Processing and Metabolite Identification

The raw data files generated from UHPLC-MS/MS were processed using Compound Discoverer 3.1 (CD3.1, Thermo Fisher) to perform peak alignment, peak picking, and quantitation for each metabolite. The main parameters were set as follows: retention time tolerance, 0.2 min; actual mass tolerance, 5 ppm; signal intensity tolerance, 30%; signal/noise ratio, 3; and minimum intensity, 100,000. After that, peak intensities were normalized to the total spectral intensity. The normalized data were used to predict the molecular formula based on additive ions, molecular ion peaks, and fragment ions. And then the peaks were matched with mzCloud (https://www.mzcloud.org/, accessed on 12 September 2023), mzVault, and MassListdatabase to obtain accurate qualitative and relative quantitative results. Statistical analyses were performed using R statistical software (R version R-3.4.3), Python (Python 2.7.6 version), and CentOS (CentOS release 6.6). When data were not normally distributed, they were standardized according to the following formula: sample raw quantitation value/( sum of sample metabolite quantitation value/ sum of QC1 sample metabolite quantitation value) to obtain relative peak areas; compounds whose CVs of relative peak areas in QC samples were greater than 30% were removed, and finally the metabolite identification and relative quantification results were obtained.

#### 2.6.4. Data Analysis

These metabolites were annotated using the KEGG database (https://www.genome.jp/kegg/pathway.html, accessed on 14 September 2023), HMDB database (https://hmdb.ca/metabolites, accessed on 14 September 2023), and LIPIDMaps database (http://www.lipidmaps.org/ accessed on 15 September 2023).

### 2.7. Statistical Analysis

The data were statistically analyzed using SPSS 26.0 (SPSS Inc., Chicago, IL, USA) and PRISM 8.3.0.538 software (GraphPad Software Inc, San Diego, CA, USA). Analysis of variance (ANOVA) was performed following Tukey’s honest significant difference (HSD) test to identify significant differences among the samples (*p* < 0.05).

## 3. Results

### 3.1. Morphology of T. harzianum TW21990 with B. vietnamiensis B418 in Co-Culture

**Figure 1:**
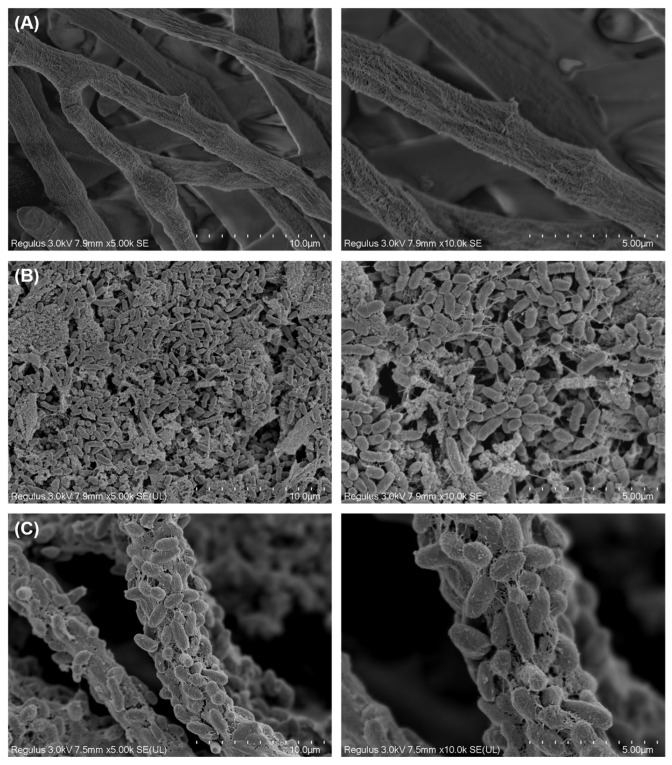
Scanning electron microscopy (SEM) images of *Trichoderma harzianum* TW21990 mycelia in monoculture (**A**), *Burkholderia vietnamiensis* B418 cells in monoculture (**B**), and B418 + TW21990 in co-culture (**C**). Scale bar: 10 μm (**left**) and 5 μm (**right**).

Scanning electron microscopy (SEM) was used to study the morphological characteristics of *T. harzianum* TW21990 and *B. vietnamiensis* B418 in a co-culture broth, where TW21990 mycelia provided a scaffold for B418 cells to grow, and B418 cells attached to the mycelia and interacted with fungi through energy dynamics and the production of bioactive secondary metabolites. As shown in Figure 1, there were no significant differences observed in the cell morphologies of TW21990 and B418 between the monocultures (Figure 1A,B) and co-culture (Figure 1C). This indicated that TW21990 and B418 were mutually compatible in MKB broth without any growth inhibition. Interestingly, we found that the rod-shaped bacteria cells adhered to the rough surface of the TW21990 mycelia and were tightly entwined together during co-culture, and a few individual B418 cells were present in the fermentation broth.

### 3.2. Antifungal Activity of Fermentation Filtrates of Mono- and Co-Cultures

#### 3.2.1. Inhibition Activity of Fermentation Filtrates on Mycelial Growth

**Figure 2:**
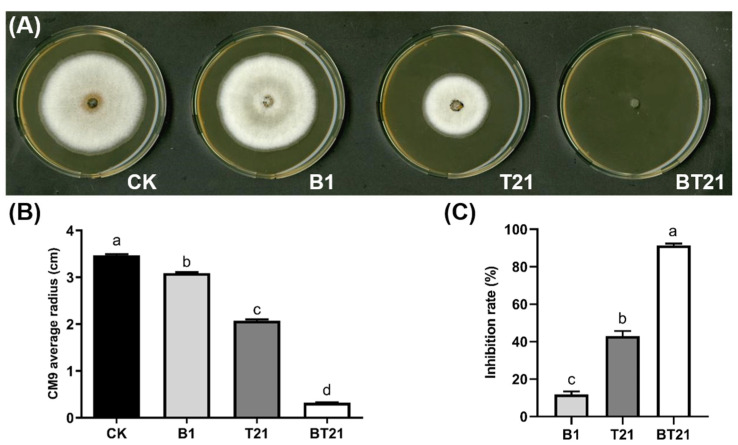
Inhibition activity of fermentation filtrates on mycelial growth of *Colletotrichum siamense* CM9 (**A**), the diameters of CM9 colonies (**B**), and inhibition rate of CM9 colony growth (**C**). Error bars represent standard errors; the letters (a to d) above the columns represent significant differences at *p* < 0.05 according to one-way ANOVA with Tukey’s HSD. CK, control with sterile distilled water; B1, fermentation filtrate of *B. vietnamiensis* B418 monoculture; T21, fermentation filtrate of *T. harizanum* TW21990 monoculture; BT21, fermentation filtrate of B418 + TW21990 co-culture.

The inhibitory activity of fermentation filtrates produced by *T. harizanum* TW21990 and *B. vietnamiensis* B418 in mono- and co-cultures against *C. siamense* CM9 was evaluated using the mycelial growth rate on the plates. As presented in Figure 2, the inhibitory effect of the fermentation filtrate of the BT21 co-culture of CM9 was significantly higher than that of B418 and TW21990 monocultures (Figure 2A,B). The inhibitory activity of the fermentation filtrates on the PDA plates was apparent in a concentration-dependent manner at 5%, 10%, and 20%. The inhibition rate of the 20% fermentation filtrate of BT21 co-culture reached 91.40%, with hardly any CM9 colonies growing on the PDA plates, whereas the inhibition rates of TW21990 and B418 monocultures were 43.02% and 11.89%, respectively (Figure 2C). These results suggest that the BT21 co-culture improved the secretion of antifungal SMs to exert a suppression effect on CM9.

#### 3.2.2. Antagonistic Activity of Fermentation Filtrates Using Detached Leaves

**Figure 3:**
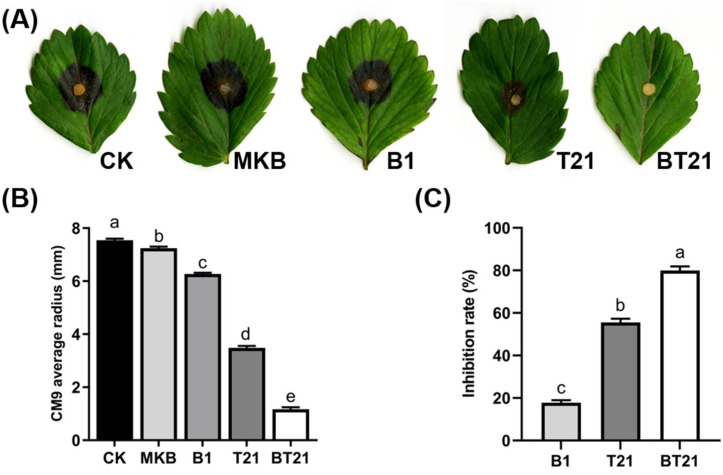
Antagonistic activity of fermentation filtrates against *C. siamense* CM9 on detached strawberry leaves (**A**), diameters of CM9 colonies (**B**), and inhibition rate of CM9 colony growth (**C**). Error bars represent standard errors; the letters (a to e) above the columns represent significant differences at *p* < 0.05 according to one-way ANOVA with Tukey’s HSD. CK, control with sterile distilled water; MKB, control with modified King’s B broth; B1, fermentation filtrate of *B. vietnamiensis* B418 monoculture; T21, fermentation filtrate of *T. harizanum* TW21990 monoculture; BT21, fermentation filtrate of B418 + TW21990 co-culture.

The antagonistic activity of the fermentation filtrates in suppressing *C. siamense* CM9 was also assessed on detached strawberry leaves. The results showed that spraying the fermentation filtrate of the BT21 co-culture reduced the severity of black spots caused by CM9 compared to spraying with SDW or the MKB broth control, with some leaves even showing no disease symptoms (Figure 3A). Accordingly, the lesion diameters of the anthracnose on the strawberry leaves treated with the fermentation filtrate of the BT21 co-culture were significantly smaller than those of the control (Figure 3B). The inhibition rate of the fermentation filtrate of the BT21 co-culture against CM9 reached 80.46%, while the inhibition rates of the TW21990 and B418 monocultures were 55.57% and 17.82%, respectively (Figure 3C). The inhibition function of the BT21 co-culture against CM9 was significantly enhanced compared to that of the monocultures and was strongly affected by the extracellular SMs.

### 3.3. Results of Strawberry Pot Experiment in Greenhouse

**Figure 4:**
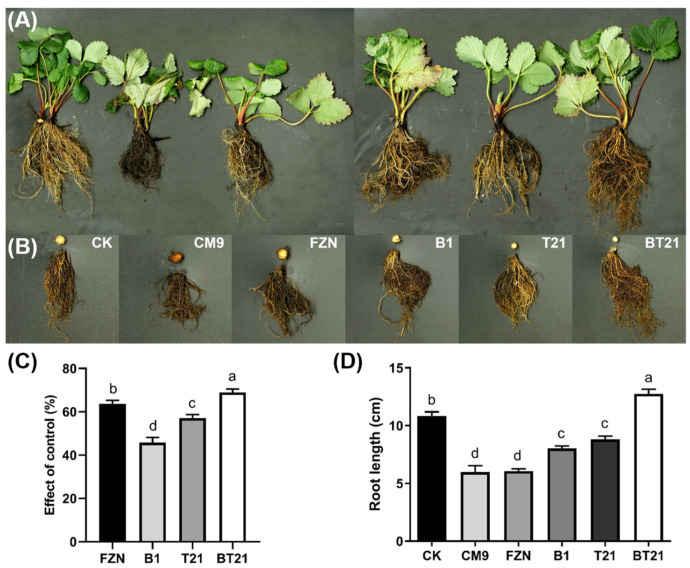
Results of strawberry pot experiment in greenhouse (**A**), cross-sections of strawberry rhizomes and roots in different treatments (**B**), control effect of different treatments (**C**), and root length in different treatments (**D**). Error bars represent standard errors; letters (a to d) above columns represent significant differences at *p* < 0.05 according to one-way ANOVA with Tukey’s HSD. CK, blank control without pathogen inoculation; CM9, negative control with *C. siamense* CM9; FZN, positive control with fungicide fluazinam; B1, *B. vietnamiensis* B418 monoculture (fermentation broth with bacteria cells); T21, *T. harizanum* TW21990 monoculture (fermentation broth with mycelia cells); BT21, B418 + TW21990 co-culture (fermentation broth with bacteria and mycelia cells).

To further investigate the biocontrol activity of the *T. harizanum* TW21990 and *B. vietnamiensis* B418 mono- and co-cultures against *C. siamense* CM9 in strawberry, a pot experiment was conducted in a greenhouse. The results showed that the strawberry seedlings in the CM9 group (negative control) developed typical anthracnose symptoms including root rot, necrotic lesions on petioles and leaves, infected rhizomes turning dark red to black, and finally whole-plant wilting (Figure 4A,B). Some of these symptoms were also observed for the strawberry plants in the other pathogen-inoculated treatments but not in the blank control group. The disease index survey revealed that the disease index of the strawberry plants treated with the BT21 co-culture (fermentation broth with bacteria and mycelia cells) was grade 1, grade 2 in the fungicide fluazinam treatment (positive control) and the TW21990 monoculture treatment (fermentation broth with mycelia cells), grade 3 for the B418 monoculture treatment (fermentation broth with bacteria cells), and grade 5 for the CM9 treatment. The control efficiency of the BT21 co-culture treatment reached 68.95%, which was followed by that of the fluazinam treatment at 63.68%, TW21990 monoculture treatment at 57.09%, and B418 monoculture treatment at 45.77% (Figure 4C). In addition, the root length and biomass of the strawberry plants treated with the mono- and co-cultures were all significantly enhanced compared to those in the CM9 control and fluazinam treatments (Figure 4B,D). The inoculation with CM9 damaged strawberry plants, which showed dramatic reductions in root length and biomass compared to those of the blank control without pathogen inoculation. The plants in the fluazinam treatment had similar root length and biomass to those in the CM9 control, despite its high control efficiency. The longest root length was measured in the BT21 co-culture treatment, with average length of 12.75 cm, which was 17.65% longer than that int he blank control. This could be attributed to the plant growth promotion and root stimulation effects of both strains in the rhizosphere, especially with the B418 monoculture treatment exhibiting more control efficiency and root promotion than expected based on its prior antifungal activity. The results revealed that the fermentation broth of the BT21 co-culture not only effectively inhibited the growth and reproduction of CM9 in soil but also promoted the root development of strawberry through the production of SMs in the co-culture and mutual interactions during co-existence in the rhizome.

#### 3.3.1. Analysis of Antioxidative Enzyme Activity in Strawberry

**Figure 5:**
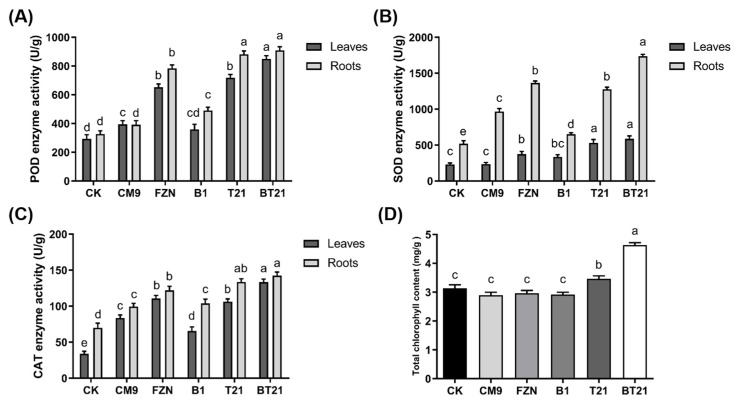
Enzyme activity and total chlorophyll content of strawberry leaves and roots. (**A**), peroxidase (POD) activity of strawberry leaves and roots; (**B**), superoxide dismutase (SOD) activity of strawberry leaves and roots; (**C**), catalase (CAT) activity of strawberry leaves and roots; and (**D**), total chlorophyll content of strawberry leaves. Error bars represent standard errors, the letters (a to e) above columns represent the significant differences at *p* < 0.05 according to one-way ANOVA with Tukey’s HSD. CK, blank control without pathogen inoculation; CM9, negative control of *C. siamense* CM9; FZN, positive control of fungicide fluazinam; B1, *B. vietnamiensis* B418 monoculture (Fermentation broth with bacteria cells); T21, *T. harizanum* TW21990 monoculture (Fermentation broth with mycelia cells); BT21, B418 + TW21990 co-culture (Fermentation broth with bacteria and mycelia cells).

Antioxidative enzymes are important components in the plant systems used for scavenging reactive oxygen species (ROS) under infection and stress conditions, which act as primary defensive enzymes in plants against phytopathogens. Superoxide dismutase (SOD) catalyzes the dismutation of superoxide radicals (O^2−^) to H_2_O_2_ and O_2_, peroxidase (POD) reduces H_2_O_2_ to H_2_O through the oxidation of phenols and amines, and catalase (CAT) converts H_2_O_2_ into less-reactive oxygen and H_2_O [31]. As shown in Figure 5A, the POD activities of the strawberry leaves and roots in the BT21 co-culture treatment were highest among all treatments, which were 1.89- and 1.78-fold higher compared to that in the blank control, respectively. The TW21990 monoculture and fluazinam treatment led to augmented POD activities of 1.44 and 1.70, being 1.22- and 1.40-fold higher compared to that of the blank control, respectively. The B418 monoculture treatment and CM9 control exhibited slight increases in POD activities compared to that of the blank control. Unlike the POD activities, the SOD activities displayed huge differences between the leaves and roots among treatments (Figure 5B). The highest SOD activities of the leaves and roots were detected in the BT21 co-culture treatment, which were 1.54- and 2.35-fold higher compared to that of the blank control, respectively. The TW21990 monoculture and fluazinam treatment resulted in improved SOD activities of 1.29 and 1.46, being 0.62- and 1.63-fold higher compared to that of the blank control, respectively. It was worth mentioning that SOD activity of strawberry roots in CM9 control increased 0.87 folds compared to blank control whereas the SOD activities of leaves in both treatments were similar. As presented in Figure 5C, CAT activities showed similar patterns to POD except CAT activity of strawberry leaves in blank control was only 48.39% to that of roots. The highest CAT activities of leaves and roots were also observed in BT21 co-culture treatment which increased 2.93 and 1.03 folds compared to blank control, respectively. Similarly to POD, CAT activities in TW21990 monoculture and fluazinam treatment exhibited increments of 2.13 and 0.91, 2.27 and 0.74 folds compared to blank control, respectively. Taken together, BT21 co-culture treatment could enhance the resistance of strawberry plants subjected to CM9 infection by improving antioxidative enzyme activities. The results of defense enzyme activity indicated that BT21 co-culture treatment increased SOD, POD, and CAT activities, thereby enhancing defense responses and improving plant resistance.

#### 3.3.2. Influence on Total Chlorophyll Content of Strawberry Leaves

The chlorophyll content is an important index to reflect plant growth and is closely related to photosynthesis and nutritional status. As shown from Figure 5D, the highest total chlorophyll content of strawberry leaves was detected in BT21 co-culture treatment with an increment of 46.14% compared to that of blank control, and TW21990 monoculture treatment also showed an improvement of 11.25%. Other treatments of B418 monoculture, fluazinam treatment, and CM9 control were slightly decreased compared to blank control, which might be caused by the adverse effects of phytopathogen inoculation. The results suggested BT21 co-culture treatment increased chlorophyll content and promoted photosynthesis.

### 3.4. Analysis of Metabolomic Profiles

#### 3.4.1. Principal Component Analysis (PCA)

To decipher the mechanism through which the improvement in the antagonistic activity against *C. siamense* CM9 of the BT21 co-culture was achieved compared with those of the TW21990 and B418 monocultures, a metabolomic analysis of the fermentation filtrates among the different culture groups was performed to elucidate SM differences among the mono- and co-cultures. In total, 478 and 795 metabolites in the BT21 co-culture were obtained and annotated in positive and negative ion modes, respectively.

**Figure 6:**
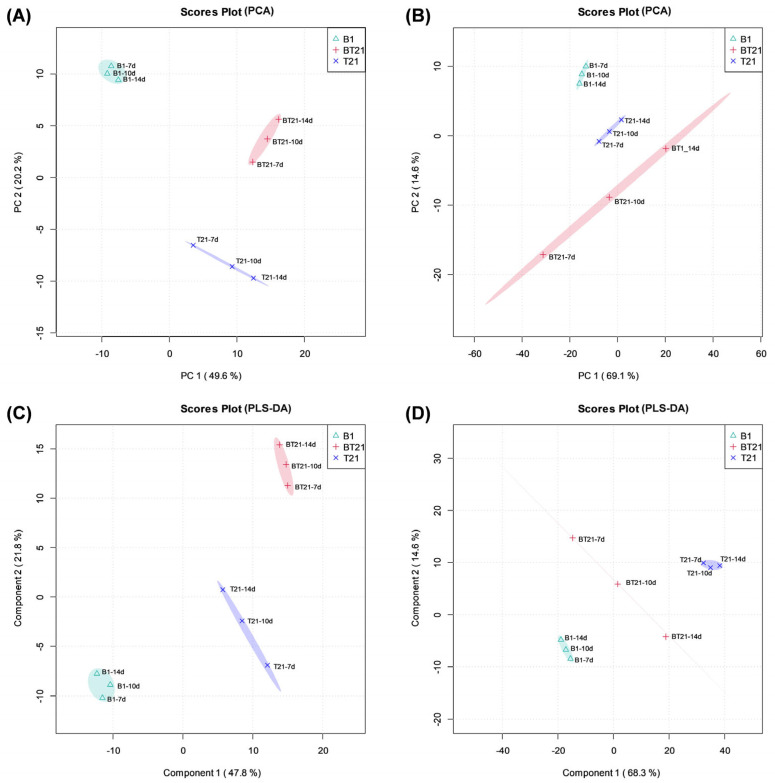
Principal components analysis (PCA) and partial least squares discriminant analysis (PLS-DA) score plots of secondary metabolites extracted from fermentation filtrates of mono- and co-cultures. (**A**) PCA score plots in positive ion mode, (**B**) PCA score plots in negative ion mode, (**C**) PLS-DA score plots in positive ion mode, and (**D**) PLS-DA score plots in negative ion mode. B1, fermentation filtrate of *B. vietnamiensis* B418 monoculture; T21, fermentation filtrate of *T. harizanum* TW21990 monoculture; BT21, fermentation filtrate of B418 + TW21990 co-culture.

Principal component analysis (PCA) is an unsupervised statistical method that can reflect the overall differences and the degree of variation in high-dimensional data while retaining the essence of their variation through different principal components [32]. In Figure 6A,B, the PCA score plots show an obvious separation trend in the metabolomic profiles among the nine samples of the B418 monoculture (B1), TW21990 monoculture (T21), and BT21 co-culture. In positive ion mode, principal component 1 (PC1) and principal component 2 (PC2) accounted for 49.6% and 20.4% of the total variability (Figure 6A), while in negative ion mode, the contribution rates of PC1 and PC2 were 69.1% and 14.6%, respectively (Figure 6B). The PCA results clearly highlighted the presence of three clusters related to two monocultures (B1 and T21) and their co-culture (BT21), which allowed the further discrimination of the metabolic composition of the co-culture from that of two monocultures.

#### 3.4.2. Partial Least Squares Discriminant Analysis (PLS-DA)

To achieve and maximize the distinction of the different groups, the samples were subjected to a supervised partial least square discriminant analysis (PLS-DA) to distinguish the datasets of each group as much as possible through the proper rotation adjustment of the principal component space [33]. As presented in Figure 6C,D, the PLS-DA score plots exhibit a significant separation trend among the co-culture (BT21) and two monocultures groups (B1 and T21), together with the excellent reproducibility of the samples within the groups. In positive ion mode, the total variance explained was 47.8% by PLS component 1 and 21.8% by PLS component 2 (Figure 6C), whereas in negative ion mode, the total variance explained was 68.3% by PLS component 1 and 14.6% by PLS component 2 (Figure 6D). The PLS-DA results effectively supported the discrimination potential of the metabolomic profiles, suggesting that the model was reliable and had good prediction ability.

#### 3.4.3. Orthogonal Partial Least Squares Discriminant Analysis (OPLS-DA)

Orthogonal partial least squares discriminant analysis (OPLS-DA) is a supervised regression modeling method in which the variation from matrix X that is not correlated to Y can be removed, thus concentrating the classification information onto one principal component [34]. OPLS-DA is employed to not only for the interpretation and classification of datasets but also for the identification of discriminant biomarkers or metabolites. Therefore, some researchers consider OPLS-DA better than PLS-DA for analysis and interpretation, although both methods possess the same predictive capability. In order to determine the discriminant biomarkers or metabolites, variable importance in the projection (VIP) analysis was performed to reflect the importance of the variables in the model regarding Y.

**Figure 7:**
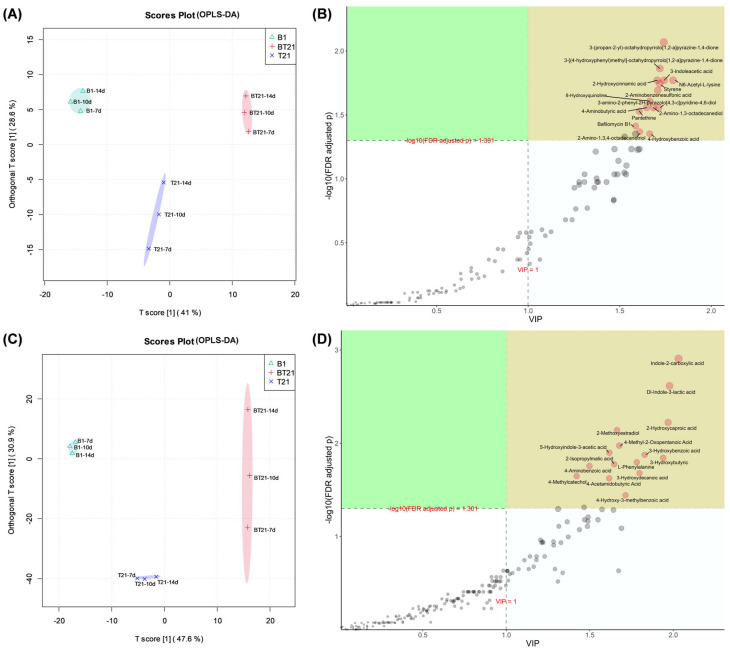
Orthogonal partial least squares discriminant analysis (OPLS-DA) of secondary metabolites extracted from fermentation filtrates of mono- and co-cultures. (**A**) OPLS-DA score plots in positive ion mode, (**B**) OPLS-DA differential metabolite diagram in positive ion mode, (**C**) OPLS-DA score plots in negative ion mode, and (**D**) OPLS-DA differential metabolite diagram in negative ion mode. B1, fermentation filtrate of *B. vietnamiensis* B418 monoculture; T21, fermentation filtrate of *T. harizanum* TW21990 monoculture; BT21, fermentation filtrate of B418 + TW21990 co-culture.

In Figure 7A, the OPLS-DA score plots of the samples from the co-culture (BT21) and two monoculture groups (B1 and T21) exhibit a significant separation trend of three confidence intervals, with scattered distribution and different contours in the positive ion mode. The cumulative R^2^X, R^2^Y, and Q^2^ were 0.696, 0.998, and 0.957, respectively, which indicated satisfactory differentiation of the mode, and the following data were reliable. The differential metabolite features among the mono- and co-cultures groups were selected using a combination of a VIP value > 1.0 and a false discovery rate (FDR) adjusted *p* value < 0.05 (Figure 7B). The differential metabolites of the BT21 co-culture in positive ion mode with significant variations were characterized accordingly and are displayed as red dots in the first quadrant (marked as yellow). There were 15 differential metabolites identified in the first quadrant (Figure 7B): 3-(propan-2-yl)-octahydropyrrolo[1,2-a]pyrazine-1,4-dione (cyclo(L-Pro-L-Val)), 3-[(4-hydroxyphenyl)methyl]-octahydropyrrolo[1,2-a]pyrazine-1,4-dione (cyclo(L-Pro-L-Tyr)), 3-indoleacetic acid (IAA), N6-acetyl-L-lysine, 2-hydroxycinnamic acid (HCA), styrene, 8-hydroxyquinoline, 2-aminobenzenesulfonic acid (2-ABSA), 3-amino-2-phenyl-2H-pyrazolo[4,3-c]pyridine-4,6-diol, 4-aminobutyric acid (GABA), 2-amino-1,3-octadecanediol, pantethine, bafilomycin B1, 2-amino-1,3,4-octadecanetrio, and 4-hydroxybenzoic acid (4-HBA).

A similar OPLSDA separation trend was observed in the negative ion mode, with cumulative R^2^X, R^2^Y, and Q^2^ values of 0.872, 0.999, and 0.961, respectively (Figure 7C). There were 15 differential metabolites identified in the negative ion mode of the BT21 co-culture in the first quadrant (Figure 7D), including indole-2-carboxylic acid (I2CA), DL-indole-3-lactic acid (ILA), 2-hydroxycaproic acid, 2-methoxyestradiol, 3-hydroxybenzoic acid (3-HBA), 5-hydroxyindole-3-acetic acid (5-HIAA), 4-methyl-2-oxopentanoic acid, 4-aminobenzoic acid (PABA), L-phenylalanine, 2-isopropylmalic acid (2-IPMA), 3-hydroxybutyric acid, 4-hydroxy-3-methylbenzoic acid (HMBA), 4-acetamidobutyric acid (N-acetyl GABA), 4-methylcatechol, and 3-hydroxydecanoic acid (3HDA).

#### 3.4.4. Univariate Analysis

**Figure 8:**
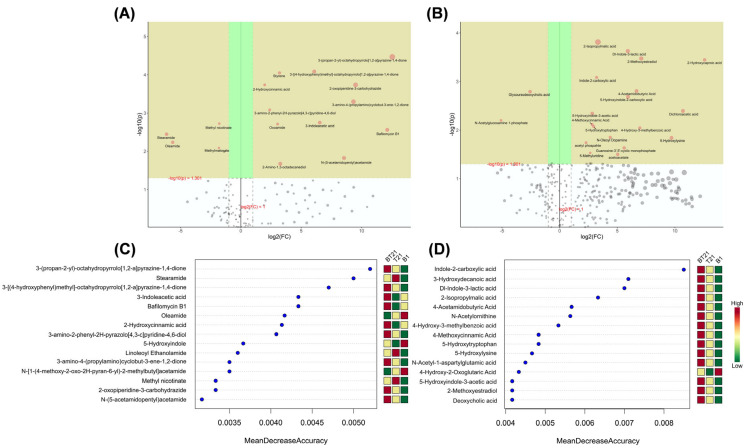
Volcano plot analysis and random forest plot of secondary metabolites extracted from fermentation filtrates of mono- and co-cultures. (**A**) Volcano plot analysis of secondary metabolites in positive ion mode, (**B**) volcano plot analysis of secondary metabolites in negative ion mode, (**C**) top 15 significant metabolites in positive ion mode identified using random forest, and (**D**) top 15 significant metabolites in negative ion mode identified using random forest. B1, fermentation filtrate of *B. vietnamiensis* B418 monoculture; T21, fermentation filtrate of *T. harizanum* TW21990 monoculture; BT21, fermentation filtrate of B418 + TW21990 co-culture.

To further assess the changes in the magnitude of the differential metabolites among different culture groups and their effects on the microorganisms, univariate analysis was performed by calculating the value of log_2_(FC), where FC is the fold change of the metabolites [35]. As shown in Figure 8A,B, the significantly different metabolites and variance were characterized by the combination of an FC value > 2.0 and a *p* value < 0.05. From the volcano plot analysis of the SMs in positive ion mode for the BT21 co-culture (Figure 8A), there were 12 upregulated compounds including cyclo(L-Pro-L-Val), cyclo(L-Pro-L-Tyr), 2-oxopiperidine-3-carbohydrazide, 3-amino-4-(propylamino)cyclobut-3-ene-1,2-dione, bafilomycin B1, 3-amino-2-phenyl-2H-pyrazolo[4,3-c]pyridine-4,6-diol, N-(5-acetamidopentyl)acetamide, IAA, 2-amino-1,3-octadecanediol, styrene, oxoamide, and HCA; and there were 4 compounds downregulated, which were stearamide, oleamide, methyl nicotinate, and methyl malonate. From the volcano plot analysis of the SMs in negative ion mode (Figure 8B), there were 18 compounds upregulated for the BT21 co-culture, including 2-IPMA, ILA, 2-methoxyestradiol, 2-hydroxycaproic acid, dichloroacetic acid (DCA), 5-hydroxylysine, N-acetyl GABA, 5-HIAA, 5-hydroxyindole-2-carboxylic acid (5-HICA), I2CA, HMBA, guanosine-3′,5′-cyclic monophosphate, acetoacetate, N-oleoyl dopamine, 5-hydroxytryptophan, 4-methoxycinnamic acid (MCA), 5-methyluridine, and acetyl phosphate; and there were 2 compounds that were downregulated, which were glycoursodeoxycholic acid (GUDCA) and N-acetylglucosamine 1-phosphate.

#### 3.4.5. Random Forest Analysis

As a nonparametric ensemble learning algorithm based on multiple binary decision trees, random forest employed the mean decrease accuracy (MDA) to measure the importance of metabolites in discriminant grouping [36]. As shown in Figure 8C, there were nine metabolites identified from the co-culture group (BT21) and six metabolites from the monoculture groups (three each from B1 and T21) in the top fifteen significant metabolites in positive ion mode. Among them, cyclo(L-Pro-L-Val), cyclo(L-Pro-L-Tyr), IAA, bafilomycin B1, and HCA displayed a great impact on the MDA in the data classification for the BT21 co-culture, while oleamide, 5-hydroxyindole, and N-[1-(4-methoxy-2-oxo-2H-pyran-6-yl)-2-methylbutyl]acetamide greatly influenced the MDA in the *B. vietnamiensis* B418 monoculture. Stearamide, linoleoyl ethanolamide, and methyl nicotinate greatly influenced the MDA in the *T. harizanum* TW21990 monoculture. As presented in Figure 8D, there were 14 metabolites identified from the co-culture group (BT21) and 1 metabolite from the B1 monoculture group in the top 15 significant metabolites in negative ion mode. Among them, I2CA, 3HDA, ILA, 2-IPMA, N-acetyl GABA, N-acetylornithine, HMBA, MCA, 5-hydroxytryptophan, and 5-hydroxylysine had a great impact on the MDA in the BT21 co-culture, while only 4-hydroxy-2-oxoglutaric acid (HOGA) greatly influenced the MDA in the *B. vietnamiensis* B418 monoculture.

## 4. Discussion

Strawberry anthracnose is a destructive disease caused by *Colletotrichum* spp. and has become the primary concern in the strawberry industry worldwide [37]. *C. siamense* CM9 was isolated and characterized from strawberry soil affected by root rot. The colonies of CM9 on the PDA medium were cottony and greyish white, with a relatively slow mycelial growth rate. In this study, the fermentation filtrates of *T. harzianum* TW21990 and *B. vietnamiensis* B418 in a co-culture (BT21) produced a remarkably higher inhibition rate against CM9 on PDA plates and strawberry leaves than the TW21990 and B418 monocultures. This indicated that the BT21 co-culture in MKB broth enhanced the production of secondary metabolites with antagonistic capabilities and growth-promoting effects. Similar results were observed with a co-culture of *T. atroviride* SG3403 and *Bacillus subtilis* 22 in which the fermented filtrate of the co-culture increased the antifungal metabolites produced against *F. graminearum* compared with those of the monoculture and enhanced the biocontrol effect of *Trichoderma* and *Bacillus* [24]. Ma et al. studied the biocontrol effect of a mixed-culture fermentation of *T. longibrachiatum* and *Bacillus amyloliquefaciens* on tomato *Fusarium* wilt, which was significantly stronger than that of the single-culture fermentation [38].

In addition, the fermentation broth of the BT21 co-culture in the pot experiment displayed a considerable control effect on strawberry root rot caused by CM9 and a growth-promoting effect on strawberry roots and leaves. The antioxidative enzyme activities of SOD, POD, and CAT in the strawberry leaves and roots were significantly improved, and total chlorophyll content of the strawberry leaves was augmented with the BT21 co-culture treatment. Yang et al. employed the liquid co-cultivation of *T. harzianum* LTR-2 and *Arthrobacter ureafaciens* DnL1-1, which produced significant promoting effects on wheat germination and radicle length compared with single-cultured LTR-2 or DnL1-1 [39]. Karuppiah et al. also reported that the co-culture fermentation of *T. asperellum* GDFS1009 and *Bacillus amyloliquefaciens* 1841 in a sequential inoculation method resulted in increased shoot and root lengths of maize and regulated the expression levels of defense genes in the roots [40].

The co-culture of different microorganisms has been proven to be a particularly successful strategy for activating the BGC expression and stimulating the SM production in microbes [41]. Metabolomic analysis is a powerful tool for SM discovery that could facilitate investigations into the fundamental purpose of SM production within microbial communities. Our UHPLC-MS/MS analysis revealed 478 and 795 metabolites among the compounds with significant variations in the BT21 co-culture in positive and negative ion modes, respectively. The PCA and PLS-DA displayed modifications of the metabolic profiles as a function of culture mode and fermentation period. The abundant SMs with significant variations in the BT21 co-culture were classified according to their structure and function: cyclic dipeptides (i.e., cyclo(L-Pro-L-Val) and cyclo(L-Pro-L-Tyr)), indole derivatives (e.g., IAA, I2CA, ILA, 5-HIAA, and 5-HICA), organic acids (e.g., GABA, N-acetyl GABA, 2-IPMA, 3-hydroxybutyric acid, and 3-hydroxydecanoic acid), benzenoids (e.g., 8-hydroxyquinoline, PABA, 2-hydroxycinnamic acid, 2-aminobenzenesulfonic acid, 3-hydroxybenzoic acid, and 4-hydroxy-3-methylbenzoic acid), amino acids (e.g., 5-hydroxytryptophan, 5-hydroxylysine, L-phenylalanine, N-acetylornithine, and N6-acetyl-L-lysine), and other compounds like 2-amino-1,3-octadecanediol, 2-methoxyestradiol, and 3-amino-2-phenyl-2H-pyrazolo[4,3-c]pyridine-4,6-diol. Studies have confirmed that these SMs present significant inhibitory effects on a variety of phytopathogens, so it is speculated that the enhancement in the inhibitory effects of the BT21 co-culture compared with those of the TW21990 and B418 monocultures was mainly associated with these metabolites.

Cyclic dipeptides (diketopiperazines) are the smallest cyclic peptides, being composed of two α-amino acids, and have a wide range of biological functions such as antioxidation, antitumor, antimicrobial, and nematicidal activities [42]. Our study identified two cyclic dipeptides, of cyclo(L-Pro-L-Val) and cyclo(L-Pro-L-Tyr), that were significantly upregulated in the BT21 co-culture, which might have contributed to the enhanced antifungal capabilities and control effects against *C. siamense* CM9 in strawberry. Meanwhile, the production of cyclic dipeptides increased as the fermentation time extended: the content of cyclic dipeptides after 14 days was notably higher than that after 7 and 10 days. Cyclic dipeptides have primarily been evaluated as the signaling molecules of quorum sensing (QS) in many studies. For instance, *Burkholderia cepacia* CF-66 produced cyclo(Pro-Phe), cyclo(Pro-Tyr), cyclo(Ala-Val), cyclo(Pro-Leu), and cyclo(Pro-Val) in both the D and L configurations [43]. Zin et al. characterized cyclo(L-Val-L-Pro) from *Streptomyces* sp. SUK 25 as showing high potency against methicillin-resistant *Staphylococcus aureus* (MRSA), which exhibited multiple antimicrobial targets against MRSA [44]. Wattana-Amorn et al. isolated cyclo(L-Pro-L-Tyr) and cyclo(D-Pro-L-Tyr) from a culture broth of *Streptomyces* sp. strain 22-4 and examined their antibacterial activities against *Xanthomonas axonopodis* and *Ralstonia solanacearum,* which showed an MIC of 31.25 μg/mL [45].

Interestingly, a myriad of indole derivatives were characterized among the compounds with significant variations in the BT21 co-culture including IAA, I2CA, ILA, 5-HIAA, and 5-HICA. It has been widely recognized that indole derivatives function as auxin phytohormones and scaffolds for various receptors by stimulating the root development and activating the immune system of plants against biotic and abiotic factors [46]. The production of and growth improvement in IAA and its derivatives have been reported in *Burkholderia heleia* PAK1-2, *B. pyrrocinia* JK-SH007, *B. phytofirmans* PsJN, *T. harzianum* WKY1, *T. viride* VKF3, and *T. atroviride* Karsten [47,48]. The high contents of indole derivatives observed in the BT21 co-culture might explain their notable growth-promoting effect on the strawberry roots and leaves in the pot experiment, especially regarding the improvement int he total chlorophyll content and defense-related enzyme activities (SOD, POD, and CAT). Similar results were reported by Wei et al. for a synthesized indole analogue D21, which increased the chlorophyll content and defensive enzyme activities in tobacco [49]. Wu et al. found that a co-culture of *T. asperellum* GDFS1009 and *Bacillus amyloliquefaciens* ACCC11060 exhibited high yields of IAA and indole-3-carboxylic acid (I3CA) with an inoculation ratio of *Bacillus*:*Trichoderma* = 1.9:1 [50]. Furthermore, some indole derivatives like IAA and ILA exhibited antioxidant and antimicrobial activities against *Salmonella* spp., *Staphylococcus* spp., *E. coli*, and *Listeria monocytogenes* [51].

There were also various organic acids, including GABA, N-acetyl GABA, 2-IPMA, 3-hydroxybutyric acid, and 3-hydroxydecanoic acid, that were significantly upregulated in the BT21 co-culture, which showed antagonistic abilities and growth-promoting effects that might have been involved in the reduction in *C. siamense* CM9 and in the root development of strawberry treated with the BT21 co-culture. For instance, GABA is commonly used as a bioactive compound in the food, pharmaceutical, and feed industries, while N-acetyl GABA is an intermediate metabolite of GABA with antioxidant and antimicrobial activities. Xiao et al. reported that GABA increased the biocontrol efficacy of *Sporidiobolus pararoseus* Y16 of the *Aspergillus* rot of grapes, which was achieved by inducing the activities of resistance-related enzymes (PPO, POD, and PAL) and the related gene expressions in grapes [52]. 2-IPMA is an essential intermediate in the biosynthesis of leucine. Ricciutelli et al. found mild antioxidant activity of 2-IPMA in wine against *E. coli*, *S. aureus*, *L. monocytogenes*, and *S. enterica* with an MIC of 409 µg/mL [53]. Zhu et al. investigated a co-culture of *T. yunnanense* SR38 and *Paenibacillus peoriae* SR235, which showed increased disease resistance activity against *F. oxysporum*; they identified DL-3-phenyllactic acid, 3-hydroxydecanoic acid, and (2S)-2-isopropylmalate as key substances, and they speculated that these components could bind to the membrane receptors of plant cells, induce plant immune response, and subsequently enhance disease resistance in *Crocus sativus* [54].

Benzenoids are another important class of organic compounds that were characterized in the BT21 co-culture that showed significant variations; we identified 8-hydroxyquinoline, PABA, 2-hydroxycinnamic acid, 2-aminobenzenesulfonic acid, 3-hydroxybenzoic acid, and 4-hydroxy-3-methylbenzoic acid. The presence of a benzene ring imparts these compounds distinct resonance stabilization and enhanced stability. The antimicrobial and antioxidant properties of 8-hydroxyquinoline against *S. aureus*, *Enterococcus faecium*, *L. monocytogenes*, and *Mycobacterium avium* have been widely reported to be involved in the inhibition of RNA synthesis and metallopeptidases [55]. PABA is a benzoic acid derivative with a broad antifungal effects including *F. graminearum*, *Rhizoctonia solani*, *Sclerotinia sclerotiorum*, and *Valsa ambiens,* even at a low concentration of 3 mM. Laborda et al. found PABA in the secretions of rhizobacterium *Lysobacter antibioticus* OH13, which showed high curative effects against infection with *Colletotrichum fructicola* in pears by inhibiting septation during cell division [56]. 2-Hydroxycinnamic acid plays an important role in regulating microbial secondary metabolism and influencing fungal reproduction, development, and adaptive response. Keman and Soyer reported that a continuous treatment of vanillic acid and 2-hydroxycinnamic acid did not induce the resistance of *S. aureus*, with MICs of 2.5 and 1.6 mg/mL, respectively [57]. The versatility of benzenoids produced under the BT21 co-culture conditions might have contributed to their antagonistic abilities.

Amino acids not only act as essential substrates for protein biosynthesis but also play crucial roles in the signaling processes in abiotic stress resistance. There were plentiful amino acids with significant variations that were identified in the BT21 co-culture like 5-hydroxytryptophan, 5-hydroxylysine, L-phenylalanine, N-acetylornithine, and N6-acetyl-L-lysine. Wu et al. compared two different inoculation ratios of *T. asperellum* GDFS1009, and *Bacillus amyloliquefaciens* ACCC11060, and they found significantly high proportions of amino acids, which were obtained in a ratio of 1:1, with eight amino acids of D-aspartic acid, L-allothreonine, L-glutamic acid, L-histidine, L-isoleucine, L-leucine, L-proline, and L-serine [50]. The metabolism of amino acids such as tyrosine and tryptophan has been shown to be involved in the plant defense response to phytopathogen infection. Tugizimana et al. reported higher levels of tyrosine, tryptophan, and 5-hydroxytryptophan; their downstream derivatives were deployed by *Paenibacillus alvei*-primed sorghum against infection by *Colletotrichum sublineolum* [58]. Deng et al. suggested that phenylalanine improved the secretion of phenylethanol, promoted the formation of *Meyerozyma caribbica* biofilms, and enhanced the biocontrol performance against jujube black spot rot caused by *Alternaria alternata* [59].

Overall, *T. harzianum* TW21990 and *B. vietnamiensis* B418 exhibited high compatibility and metabolic complementation under co-culture conditions. The BT21 co-culture system enhanced the antagonistic activities in strawberries and showed growth-promoting effects through increased SM production. A metabolomic analysis of fermentation filtrates was performed to elucidate differences in SMs among mono- and co-cultures. Although various kinds of abundant SMs that significantly differed between the mono- and the co-cultures were identified, their antagonistic and growth-promoting effects need to be further validated using the corresponding standard compounds. Our results provide a basis for better understanding the complex metabolic changes associated with fungi–bacteria interactions in co-cultures.

## 5. Conclusions

The co-culturing of microbes beneficial for plant is an efficient strategy for stimulating the production of secondary metabolites to achieve improvements in antagonistic activities and growth-promoting effects. The fermentation broth of *Trichoderma harzianum* TW21990 and *Burkholderia vietnamiensis* B418 produced an improved control efficiency against strawberry root rot caused by *Colletotrichum siamense* CM9 and enhanced the root development, antioxidative enzyme activities, and total chlorophyll contents of strawberry. UHPLC-MS/MS analysis of fermentation filtrates demonstrated that the abundant SMs that significantly varied in the BT21 co-culture, including cyclic dipeptides, indole derivatives, organic acids, benzenoids, and amino acids, were associated with the enhancement produced by the BT21 co-culture compared with the TW21990 and B418 monocultures. The results of this study provide theoretical and data support for further development of biocontrol agents.

## Figures and Tables

**Table 1:** Experimental treatments in strawberry pot experiment in greenhouse.

Number	Designation	Treatment
1	CK	Blank control without pathogen inoculation
2	CM9	10 mL *Colletotrichum siamense* CM9 suspension (1 × 10^6^ cfu/mL)
3	FZN	10 mL CM9 suspension (1 × 10^6^ cfu/mL) + 10 mL fungicide fluazinam 10^−3^ dilution (Shirlan, Syngenta)
4	B1	10 mL CM9 suspension (1 × 10^6^ cfu/mL) + 10 mL 14-day *B. vietnamiensis* B418 monoculture (fermentation broth with bacteria cells)
5	T21	10 mL CM9 suspension (1 × 10^6^ cfu/mL) + 10 mL 14-day *T. harizanum* TW21990 monoculture (fermentation broth with mycelia cells)
6	BT21	10 mL CM9 suspension (1 × 10^6^ cfu/mL) + 10 mL 14-day B418 + TW21990 co-culture (fermentation broth with bacteria and mycelia cells)

## Data Availability

The data related to this paper may be requested from the corresponding author.
